# Early detection and staging of retinitis pigmentosa using multifocal electroretinogram parameters and machine learning algorithms

**DOI:** 10.1007/s13246-025-01577-3

**Published:** 2025-06-16

**Authors:** Bayram Karaman, Ayse Öner, Aysegül Güven

**Affiliations:** 1 Graduate School of Natural and Applied Sciences, Biomedical Engineering Graduate Program, Erciyes University, Kayseri, Turkey; 2 Department of Electrical and Electronics Engineering, Engineering and Architecture Faculty, Tokat Gaziosmanpasa University, Tokat, Turkey; 3 Department of Ophthalmology, Acibadem Taksim Hospital, Istanbul, Turkey; 4 Department of Biomedical Engineering, Engineering Faculty, Erciyes University, Kayseri, Turkey

**Keywords:** Retinitis pigmentosa, Multifocal electroretinogram, Support vector machine, Naive Bayes, Logistic regression, Multilayer perceptron neural networks

## Abstract

Retinitis pigmentosa is an inherited retinal disease caused by damage to photoreceptor cells. Diagnosis and staging of this disease are crucial for early intervention and effective treatment planning. In this study, the amplitude and latency features of N1, P1, and N2 waves obtained from multifocal electroretinogram responses over five rings were used with binary and multiclass classification methods using four different machine learning algorithms to distinguish retinitis pigmentosa patients from healthy individuals and to evaluate the stages of the disease. Binary classifications were performed for six different groups, and the Naive Bayes (NB) algorithm performed the best on all evaluation metrics, achieving 99% accuracy in distinguishing healthy individuals from each disease stage. Furthermore, multiclass classification was applied in two different steps. In the first step, the Naive Bayes model achieved 82% accuracy in four-class classification, including healthy individuals. Considering the near-perfect separability of healthy individuals, in the second step, a three-class classification including only disease stages was performed, and the model achieved 76% accuracy. These results indicate that the proposed approach provides objective and accurate staging for retinitis pigmentosa and can serve as a valuable decision support system to assist ophthalmologists in clinical practice.

## Introduction

Retinitis pigmentosa is a type of inherited retinal disease caused by damage to photoreceptor (cone and rod) cells in the retina. This disease affects approximately one in 3–4 thousand people worldwide [1–3]. In Türkiye, it is estimated to affect 15,000–20,000 people due to the prevalence of consanguineous marriages [4]. This disease has a long-term impact on individuals, typically progressing over a period of 10 to 30 years. It consists of three stages: early, mid, and advanced. Early-stage patients may have decreased night vision and narrowing of peripheral vision. In mid-stage patients, the decrease in night vision is more pronounced than in the early stage, and patients notice a decrease in peripheral vision. Finally, advanced-stage patients lose their central visual field [5]. Currently, there is no definitive cure for the disease, but there are some approaches to delay the progression of the disease. These approaches vary at different stages of the disease. In patients with retinitis pigmentosa, pharmacologic treatments in early-stage, stem cell therapies in mid-stage, and gene therapy in advanced-stage can help delay disease progression [6–8]. These treatment modalities can potentially reduce the effects of retinitis pigmentosa and preserve visual function. Therefore, it is crucial to accurately identify the stages of the disease to improve the quality of life of individuals with retinitis pigmentosa and to determine appropriate treatment strategies.

In recent years, imaging and electrophysiologic techniques such as Optical Coherence Tomography Angiography (OCTA) [9], Electroretinogram (ERG) [10], Visual Field (VF) [11], Visual Acuity (VA) [11], and Multifocal Electroretinogram (MfERG) [12] have been widely used by ophthalmologists in the evaluation of retinitis pigmentosa. Specifically, the MfERG technique is crucial because it allows both the extraction of electrophysiologic information and the topographic mapping of different regions of the retina. In this way, the effects of the disease on different regions of the retina and the monitoring of disease progression can be analyzed in more detail. The MfERG technique usually divides the retina into 61 or 103 hexagonal sectors, according to International Society for Clinical Electrophysiology of Vision (ISCEV) standards. These hexagonal regions are rapidly stimulated using a pseudo-random black/white sequence (m-sequence), generating individual responses for each hexagon. Standard MfERG responses (first-order responses) consist of three fundamental waves. The first of these waves is the N1 wave, which is an initial negative deflection; the second is the P1 wave, which occurs after the negative deflection and has a maximum positive peak; and finally, the N2 wave, which is the second negative wave after P1 [13].

Previous studies have noted external retinal electroretinographic changes in retinitis pigmentosa patients, but in all of these studies, the amplitudes and latencies of MfERG responses were analyzed by statistical methods, and the results differed fundamentally [12, 14–18]. In this study, we aimed to distinguish individuals with retinitis pigmentosa from healthy individuals and to classify the stages of the disease by practically extracting the amplitude and latency features of N1, P1, and N2 waves from MfERG responses.

Retinitis pigmentosa is a multiclass disease, and accurate staging of this disease is crucial for early intervention that can delay or prevent its progression. However, the staging of disease is often based on clinical examinations and doctors’ experience. This can lead to misdiagnosis of stages and unnecessary waste of time. Therefore, it is becoming increasingly important to use an effective decision support system to assist ophthalmologists with an accurate and rapid diagnosis. These decision support systems can help predict its stages by evaluating objective data and using machine learning algorithms.

The number of studies using machine learning techniques to diagnose and stage retinitis pigmentosa is extremely limited in the literature. The scarcity of such studies suggests that its diagnosis and staging are still based on more traditional methods in the clinic. However, recent studies on retinitis pigmentosa using these techniques have generally focused on early detection or segmentation. We briefly review these studies here. Chen et al. presented pre-trained Inception Resnet V2, Xception, and Inception V3 deep learning-based transfer learning methods to classify retinitis pigmentosa patients and control groups using color fundus images. They found that the highest classification accuracy among these three transfer learning methods is the Xception method, with 96.00% [19]. To detect the presence of retinitis pigmentosa, Masumoto et al. used ultra-widefield pseud color (UWPC) and ultra-widefield autofluorescence (UWAF) images and evaluated the performance of convolutional neural networks. As a result of their experiments, they obtained 99.1% and 99.3% specificity and sensitivity for UWPC images and 99.5% and 100% for UWAF images, respectively [20]. Iadanza et al. extracted features from pupillometry data to diagnose retinitis pigmentosa disease in pediatric individuals. They classified these features using two support vector machine (SVM) algorithms, one for each eye. Subsequently, they combined the two SVM algorithms into an ensemble model and achieved an accuracy of 84.6% [21]. On the other hand, Yassin et al. investigated the superimposition of different multimodal images using manual alignment and Artificial Intelligence (AI) in individuals with retinitis pigmentosa. They used infrared images from microperimetry, spectral domain OCT and near infrared images from a scanning laser ophthalmoscope. As a result of this study, it was determined that AI aligned infrared images more successfully than the manual alignment method in retinitis pigmentosa patients [22]. Wang et al. proposed two deep learning-based models, U-Net and sliding window (SW), for automatic segmentation of retinal layer thicknesses using B-scans of spectral domain OCT of individuals with retinitis pigmentosa. They then combined these models to create a hybrid model. They found that this hybrid model has superior performance compared to U-Net and SW in measuring the retinal layer thickness in OCT images of retinitis pigmentosa patients [23]. In another study, Arsalan et al. developed RPS-Net, a deep learning-based semantic segmentation network specifically designed to detect retinal pigment markers automatically and retinitis pigmentosa detection using fundus images. They reported that this deep learning network provides superior segmentation performance compared to traditional deep learning models [24]. As seen in these papers, previous studies have focused on detecting the presence of retinitis pigmentosa disease using machine learning and accurately classifying the associated structures. However, there are no studies on its staging using MfERG techniques and machine learning algorithms.

In addition, two studies in the literature performed retinitis pigmentosa staging based on manual methods. When we look at these studies, a study conducted by Iftikhar and colleagues aimed to classify the severity of retinitis pigmentosa disease. In this study, VF width, VA, and spectral domain OCT ellipsoid zone parameters were used. These parameters were scored on a scale of 0–5 based on the distribution of the disease, creating a scoring criterion. A cumulative criterion (0–15) was obtained to grade individuals with retinitis pigmentosa from zero to five. As a result of the study, individuals with retinitis pigmentosa were divided into five groups based on cumulative criteria, and the scores associated with the methods were found to be statistically significant when compared to each other [25]. In another study, Öner et al. classified the severity of the disease similarly to the method applied by Iftikhar et al. by adding MfERG responses P1 amplitudes to VA, VF (diameter), and OCT (ellipsoid zone width) tests. As a result, in this study, it has been stated that the stages of retinitis pigmentosa are significantly associated with scores related to the parameters of the tests. As understood from the above-mentioned studies, its staging has been performed manually, employing multiple techniques in this process [26]. In this study, by combining the amplitudes and latencies of the N1, P1 and N2 waves of MfERG responses, we used machine learning classifiers to both automatically distinguish individuals with retinitis pigmentosa from healthy and automatically classify it into its stages.

However, various machine learning classifiers have been used many times in the literature to stage diseases such as Alzheimer’s [27], Parkinson’s [28], Diabetic Retinopathy [29], and Obesity [30]. It has been reported that these classifiers have achieved successful results in staging these diseases. Among these, the most used classifiers are Support Vector Machines (SVM) and Multilayer Perceptron Artificial Neural Network (MLP-ANN). In the current study, in addition to these classifiers, Logistic Regression (LR) and Naive Bayes (NB) classifiers, which give successful results in binary classification, were applied and compared (Figs. 10–15).

The main objective of this study was to help ophthalmologists develop an effective method that automatically diagnoses individuals with retinitis pigmentosa disease and classifies them into disease stages. To achieve this goal, amplitude and latency features were extracted from MfERG responses. Then, these features were used as feature vectors in SVM, NB, LR and MLP-ANN algorithms for binary and multiclass detection of retinitis pigmentosa.

## Materials and methods

**Fig. 1:**
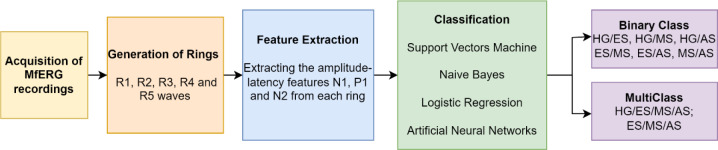
Block diagram of the proposed work (Healthy Group-HG; Early Stage-ES; Mid Stage-MS; Advanced Stage-AS)

The approach proposed in this study consists of four main stages: MfERG data collection, ring structure generation, feature extraction, and classification. A comprehensive illustration of this configuration is shown in Fig. 1, and the subsequent subsections go into great depth on the steps.

### Description of the data

All study procedures were conducted in accordance with the Declaration of Helsinki and approved by the Acıbadem Mehmet Ali Aydınlar University Medical Research Evaluation Board (ATADEK-2023-16/553). The data used for this study were obtained retrospectively from the Department of Ophthalmology, Acıbadem Kayseri Hospital, and the diagnosis of retinitis pigmentosa was confirmed using ocular history, fundus photographs, VF, OCT, and International Society for Clinical Electrophysiology of Vision (ISCEV) [13] standardized MfERG and ERG tests. The study included 98 eyes of 77 patients with retinitis pigmentosa and 34 eyes of 19 healthy individuals. In line with the inclusion criteria, individuals with typical findings on fundus examination, consistent loss on the Humphrey 30 − 2 visual field test, and the diagnosis of the disease supported by the MfERG test were recruited; however, individuals with additional eye defects such as uveitis, cataract, and glaucoma were excluded. Due to the use of retrospective data in the study, previous staging results with VA, VF, OCT, and MfERG modalities were reviewed by an expert ophthalmologist, and patients were divided into three groups: early stage (33 eyes of 19 individuals), mid stage (31 eyes of 27 individuals), and advanced stage (34 eyes of 32 individuals). To prevent possible bias in the evaluation process, a blind evaluation was performed by a physician and the physician performed an objective evaluation without knowing which group the signals belonged to. With this method, bias in the clinical evaluation process was minimized, and a more reliable classification was achieved. Table 1 presents the demographic and clinical characteristics of the individuals who participated in the study.

**Table 1:** Demographic characteristics of healthy group and retinitis pigmentosa patients

Subjects	Total Subjects (Number of eyes)	Male/Female	Age (Mean±SD)
HG	19 (34)	13/6	35.5$\:\pm\:$13.8
ES	19 (33)	8/11	39.8$\:\pm\:$20.3
MS	27 (31)	17/10	35.9$\:\pm\:$14.8
AS	32 (34)	23/9	38.6$\:\pm\:$15.9

Healthy Group-HG; Early Stage-ES; Mid Stage-MS; Advanced Stage-AS

### Acquisition of MfERG recordings

MfERG recordings were obtained using the Metrovision MonPackOne system (France) according to ISCEV [13] procedures. Subjects were kept in the test room to adjust to the light and then their pupils were dilated. After topical anesthesia, a contact lens electrode was placed on the numb pupils for recording. The screen of the MonPackOne stimulus system was positioned 33 cm from the subject. The stimulator is composed of 61 hexagon sectors. The luminance of each hexagonal sector was independently set to 100 cd/m2 for white hexagons and 1 cd/m2 for black hexagons. The stimulus frequency was fixed at 17 Hz [31–33], and the frame rate of the system is set to 75 Hz in accordance with ISCEV standards [13, 34]. The sampling rate was 960 Hz, and each MfERG response length was 96 samples per 100 ms. To ensure signal reliability, at least 5000 responses were recorded from each eye of the participants, with noise levels carefully maintained below 5 kiloohms.

### Generation of rings and feature extraction

**Fig. 2:**
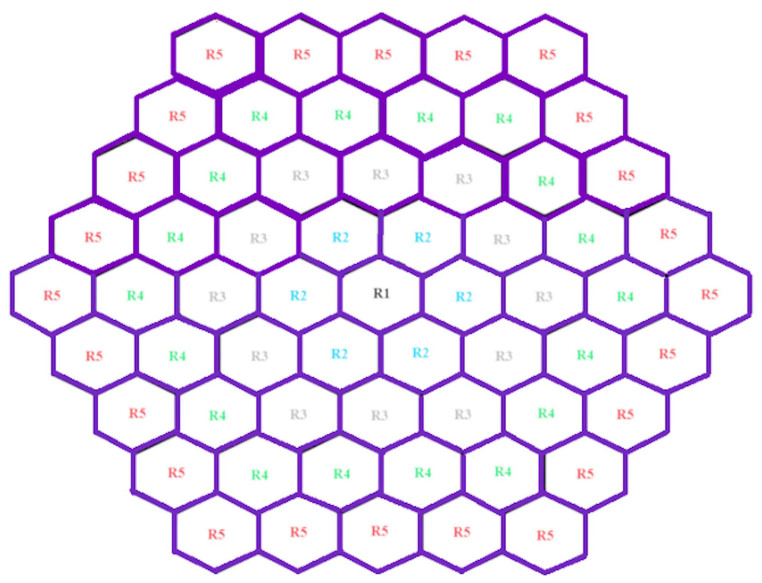
61 hexagonal sectors

**Fig. 3:**
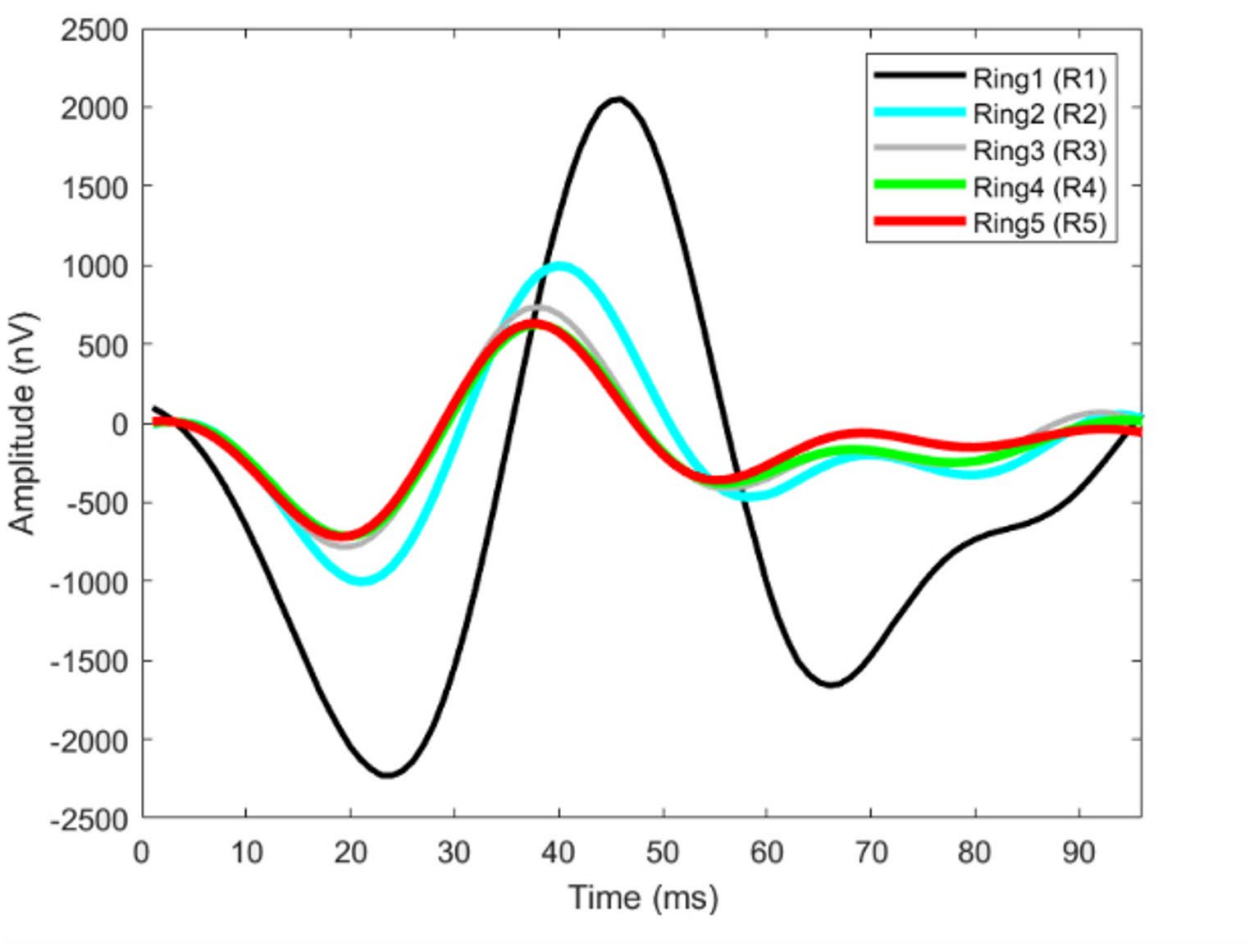
Rings (R1, R2, R3, R4, and R5) obtained by averaging from each region (MfERG response)

In this study, the first-order MfERG kernel was used. According to ISCEV standards, 61 hexagon sectors were grouped into five rings from the center to the periphery as Ring1 (R1-1 hexagon), Ring2 (R2-6 hexagon), Ring3 (R3-12 hexagon), Ring4 (R4-18 hexagon), and Ring5 (R5-24 hexagon). The position of these rings was shown on Fig. 2. Their average responses were obtained (Fig. 3). The amplitudes and latencies of the N1, P1 and N2 waves of each ring were determined using the findpeaks function developed by Matlab (R2023a-trial version). The N1 wave, usually a negative peak, was calculated by finding the minimum value within the temporal window between 9 and 32 ms. The P1 wave is followed by a positive peak and is obtained by determining the maximum value in the range of 32–50 ms. The N2 wave is the second negative peak after P1 and was calculated by finding the minimum value in the range of 50–70 ms. In total, 30 features of 15 amplitudes and 15 latencies were extracted. These features were used for the feature vector of the classification algorithms [34, 35].

**Fig. 4:**
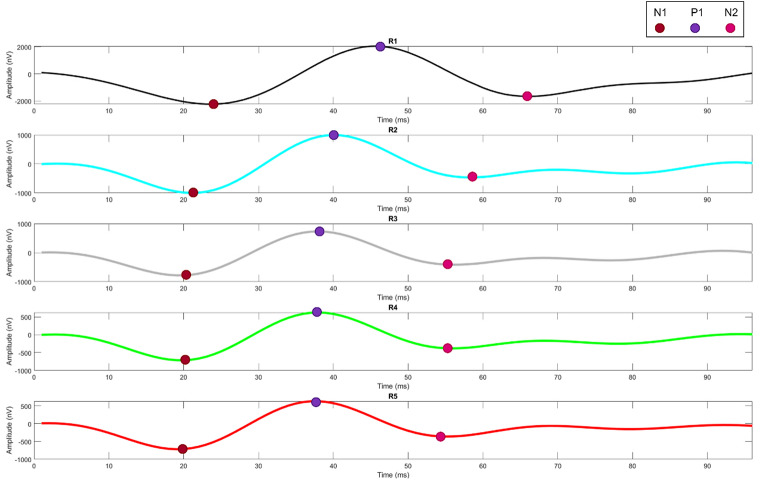
N1, P1, and N2 features extracted from each ring

Figure 4 presents an example of the parameters N1, P1, and N2 of the rings. Table 2 shows the N1, P1, and N2 amplitude and latency values of the MfERG responses of some subjects.

**Table 2:** N1, P1, and N2 amplitudes (µV) and latencies (ms) of randomly selected subjects

Amplitude and Latency Features of Rings		HG			ES			MS			AS		
		**Subject Numbers**			**Subject Numbers**			**Subject Numbers**			**Subject Numbers**		
		**2**	**15**	**23**	**4**	**12**	**21**	**6**	**19**	**26**	**9**	**17**	**29**
N1 (a)	**R1**	-990	-1162	-1237	-512	-440	-441	-486	-642	-489	-439	-482	-317
	**R2**	-712	-712	-768	-342	-225	-175	-220	-290	-220	-135	-123	-94,6
	**R3**	-616	-579	-811	-244	-266	-204	-50,9	-152	-113	-31	-127	-147
	**R4**	-717	-645	-751	-218	-239	-176	-70,1	-63,4	-15,6	-24,8	-13,1	-42,6
	**R5**	-683	-615	-761	-189	-224	-218	-23,2	-49,9	-5,7	-17,2	-62,3	-35,7
P1 (a)	**R1**	1384	1653	1273	554	662	768	621	1289	548	341	606	546
	**R2**	1306	1264	1403	504	484	376	173	456	155	158	40,9	123
	**R3**	1185	1073	1309	427	516	459	24,8	156	189	90,8	55,3	148
	**R4**	1293	1150	1444	391	500	493	99,1	106	115	74,6	105	39,8
	**R5**	1407	1253	1678	343	509	541	51,3	49,6	89,3	34,7	76,3	47,5
N2 (a)	**R1**	-1023	-1436	-926	-584	-778	-713	-620	-1292	-610	-288	-344	-536
	**R2**	-1017	-986	-1015	-367	-462	-459	-167	-348	-109	-173	-129	-190
	**R3**	-1072	-901	-983	-309	-428	-376	-59,6	-157	-201	-59,5	-18,2	-61
	**R4**	-1090	-985	-1138	-385	-457	-447	-68,7	-89	-144	-28,9	-158	-78
	**R5**	-1177	-1152	-1492	-270	-479	-468	-30,6	-44,5	-94	-19,8	-78	-73,9
N1 (l)	**R1**	24,8	28	24,8	20,5	26	29,7	19,9	31,9	33,6	40,9	23,6	41,2
	**R2**	23,8	26,4	26,5	22,8	25,3	27,4	35	28	29,7	25,8	21,1	31,6
	**R3**	24,8	26,8	26,3	25,9	25,1	26,9	28,7	32,7	29,3	44,7	47,5	32,8
	**R4**	25	26,1	27	31	23,9	25,4	30,7	14,8	19,7	29,9	35,1	45,4
	**R5**	25,2	25,3	27,6	33,4	24,5	25,9	26,9	36,6	42,5	46,8	32,4	18,2
P1 (l)	**R1**	45,4	51,5	50,8	54,7	53,2	53,4	56,3	53,7	51,8	55,4	67,6	56,9
	**R2**	43,6	45,3	47	50,8	43,9	44,8	54	47,6	69,3	65,5	32,8	51,5
	**R3**	43,4	44,7	45,3	46,3	42,8	44	55,3	49,6	43,8	61	63,4	68,6
	**R4**	43,2	44,5	45,5	49,8	41,3	42,4	55,8	54,7	33,5	61,9	54,5	69,3
	**R5**	43,5	43,6	46	53,5	42,5	43,8	67,9	58,5	59,7	67,2	56,8	56,8
N2 (l)	**R1**	71,5	72	71,4	74,5	74,5	71,3	76,3	74,9	69	71,4	84,6	89,9
	**R2**	64,6	63,8	67,5	70,9	63,6	64,7	70,4	69,4	83,5	89,6	86,4	87,6
	**R3**	62,5	62,4	63,5	69,9	60,8	60,8	72,2	65,3	58,7	74	73,4	81,4
	**R4**	61,3	62,4	63,9	68,1	58,6	59,4	77,5	74,4	48,7	73	87,8	88,8
	**R5**	61,6	61,2	64,1	73,1	60,3	60,9	80,5	77,7	74,2	77	77,4	74,3

Healthy Group-HG; Early Stage-ES; Mid Stage-MS; Advanced Stage-AS; a = amplitude; l = latency

### Classification algorithms

After extracting the features from the MfERG responses with the method mentioned above, SVM, NB, LR and MLP-ANN classifiers were used to evaluate the binary and multiclass results. The algorithms used are briefly described below.

#### Support vector machines (SVM)

SVM is an machine learning algorithm developed by Vapnik and Cortes [36] that is particularly effective for binary classification problems. This algorithm is mainly used to discriminate data points belonging to two classes by an optimal hyperplane. To find this optimal hyperplane, SVM applies the maximum margin principle. Margin refers to the distance between the support vectors, and the maximum margin is achieved when creating the best separation plane between two classes.

Different kernel functions are used depending on whether the features are linear. The Linear kernel function is preferred if the features can be linearly decomposed. However, if the features cannot be linearly decomposed, methods such as Polynomial, Gaussian, and Sigmoid kernel functions are used. These different kernel functions increase the flexibility of SVM, allowing better results for various data structures and classification problems. Figure 3 shows a linear kernel function’s support vectors and optimal hyperplane. A linear hyperplane is expressed as in Eq. (1).

1 $$\:{w}^{t}.{x}_{j}+b=0.$$

Where $\:j$ is the number of instances (N) in the dataset, $\:{x}_{j}$ is the features in the hyperplane, $\:w$ is the weight vector, and $\:b$ is the bias value of the hyperplane. The hyperplane if the dataset consists of a linearly separable structure with class labels $\:y\in\:\:\{1,-1\}$:

2 $$\:{w}^{t}.{x}_{j}+b\ge\:1,\:\:y=1$$

3 $$\:{w}^{t}.{x}_{j}+b\le\:-1,\:y=-1.$$

is expressed as. Combining the entire dataset, i.e., Eq. (2) and Eq. (3), results in Eq. (4)

4 $$\:{y}_{j}\left({w}^{t}.{x}_{j}+b\right)\ge\:1\:\:\:\:\:\:\:j=\text{1,2},\text{3,4},\dots\:.n\:\:.$$

**Fig. 5:**
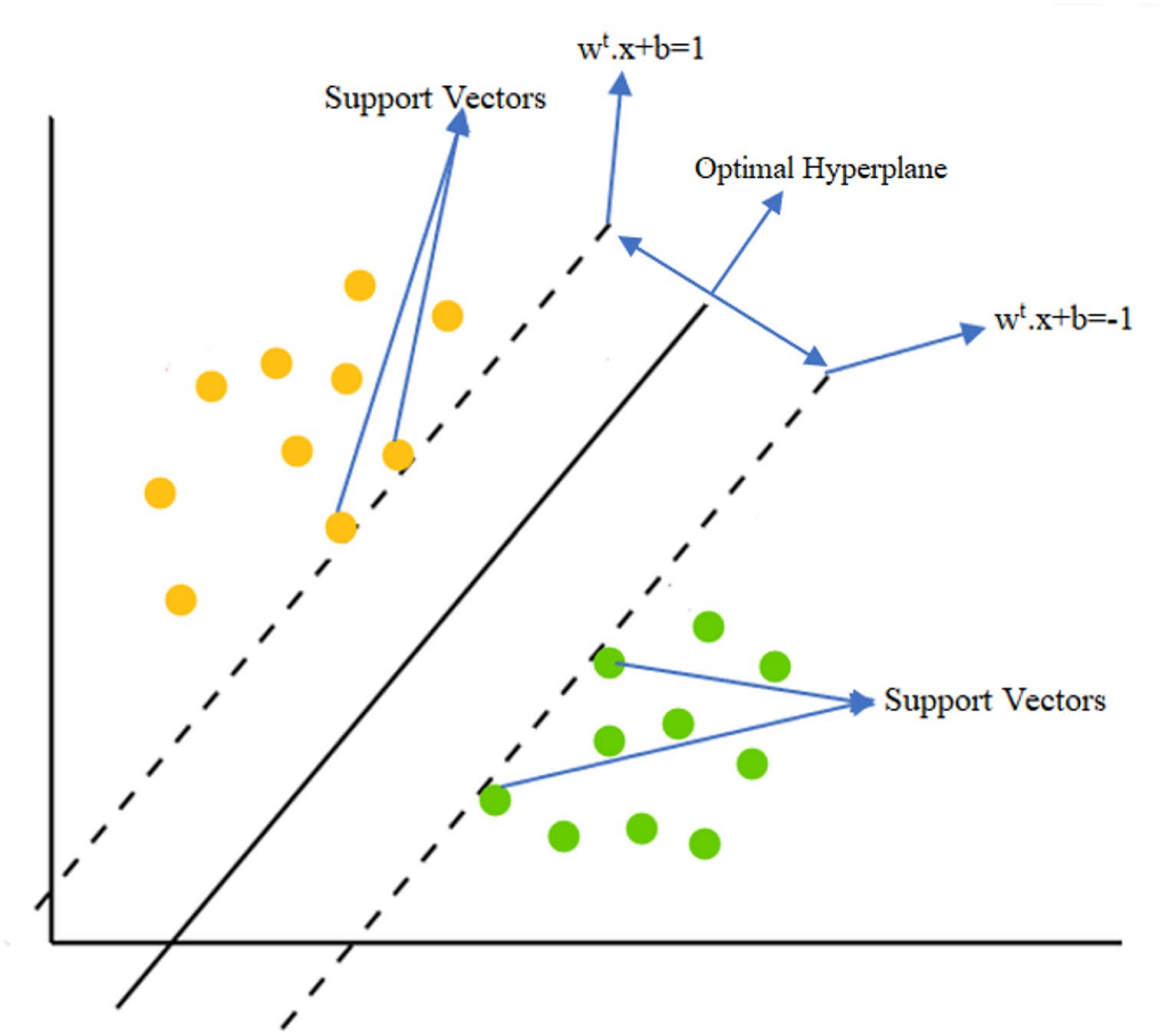
Support vectors and optimal hyperplane of the linear kernel function

The optimal hyperplane is between the support vector points and parallel lines, as shown in Fig. 5. The margin of this hyperplane is expressed as $\:2/\Vert\:w^2$‖. Then, using the Lagrange coefficients ($\:\lambda\:$), the optimal hyperplane, and Eq. (4), the decision rule in Eq. (5) is obtained.

5 $$y = f\left( x \right) = sign(\sum\nolimits_j {{\lambda _j}{y_j}\langle x.{x_j}\rangle + b} ).$$

#### Naive Bayes (NB)

NB, based on Bayes’ theorem, is a simple machine learning algorithm used to solve classification problems. As a basic principle, the NB algorithm performs probabilistic classification by treating the features in the dataset independently. That is, it works under the assumption that each feature is evaluated independently of the other features in class determination. In this way, the probabilities of the features belonging to the classes are calculated, the class with the highest probability is assigned to the data point, and classification is performed [37]. The mathematical calculation of the NB algorithm is given in Eq. (6):

6 $$\:P\left(K/L\right)=\frac{P(L/K)P\left(K\right)}{P\left(L\right)}.$$

Where $\:P\left(K\right)$ is the probability of $\:K;\:P\left(L\right)$ is the probability of $\:L;\:P(K/L)$ is the probability of $\:K$ based on $\:L$; $\:P(L/K)$ is the probability of $\:L$ based on $\:K$.

#### Logistic regression (LR)

LR [38] is an algorithm that establishes a polynomial equation between multiple independent variables and a dependent variable and is used to classify data. This algorithm uses a sigmoid function, shown in Eq. (7), which estimates the probability values of the dependent variable between 0 and 1.

7 $$\:{h}_{\theta\:}\left(x\right)=\frac{1}{1+{e}^{-({\theta\:}_{0}+{\theta\:}_{1}.x1)}}.$$

In this equation, $\:\theta\:$ represents the weights, and $\:x$ represents the number of features in the dataset. A cost function is usually used when the LR algorithm learns the weights according to the dataset. The cost function is a function that evaluates the accuracy of the learned weights and measures the performance of the algorithm. The LR algorithm minimizes the cost function to obtain the best weights. This function is expressed in Eq. (8).

8 $$\:J\left(\theta\:\right)=\frac{1}{m}\left[\sum\:_{i=1}^{m}(-{y}^{i}.\text{log}\left({p}_{\theta\:}\left({x}^{i}\right)\right)-\left(1-{y}^{i}\right).\text{log}(1-{p}_{\theta\:}\left({x}^{i}\right))\right].$$

Where $\:m$ represents the amount of data in the dataset, and $\:y$ represents the actual class labels. The LR model training is completed once the cost function reaches the optimal value. Then, the probability values, the model outputs, are compared with the threshold values to predict the classes according to Eq. (9) and Eq. (10).

9 $$\:If\:h\_\theta\:\:\left(x\right)<0.5\:;y\:output\:value\:0$$

10 $$\:If\:h\_\theta\:\:\left(x\right)\ge\:0.5,\:the\:output\:value\:of\:y\:is\:1\:.$$

#### Multilayer perceptron artificial neural network (MLP-ANN)

**Fig. 6:**
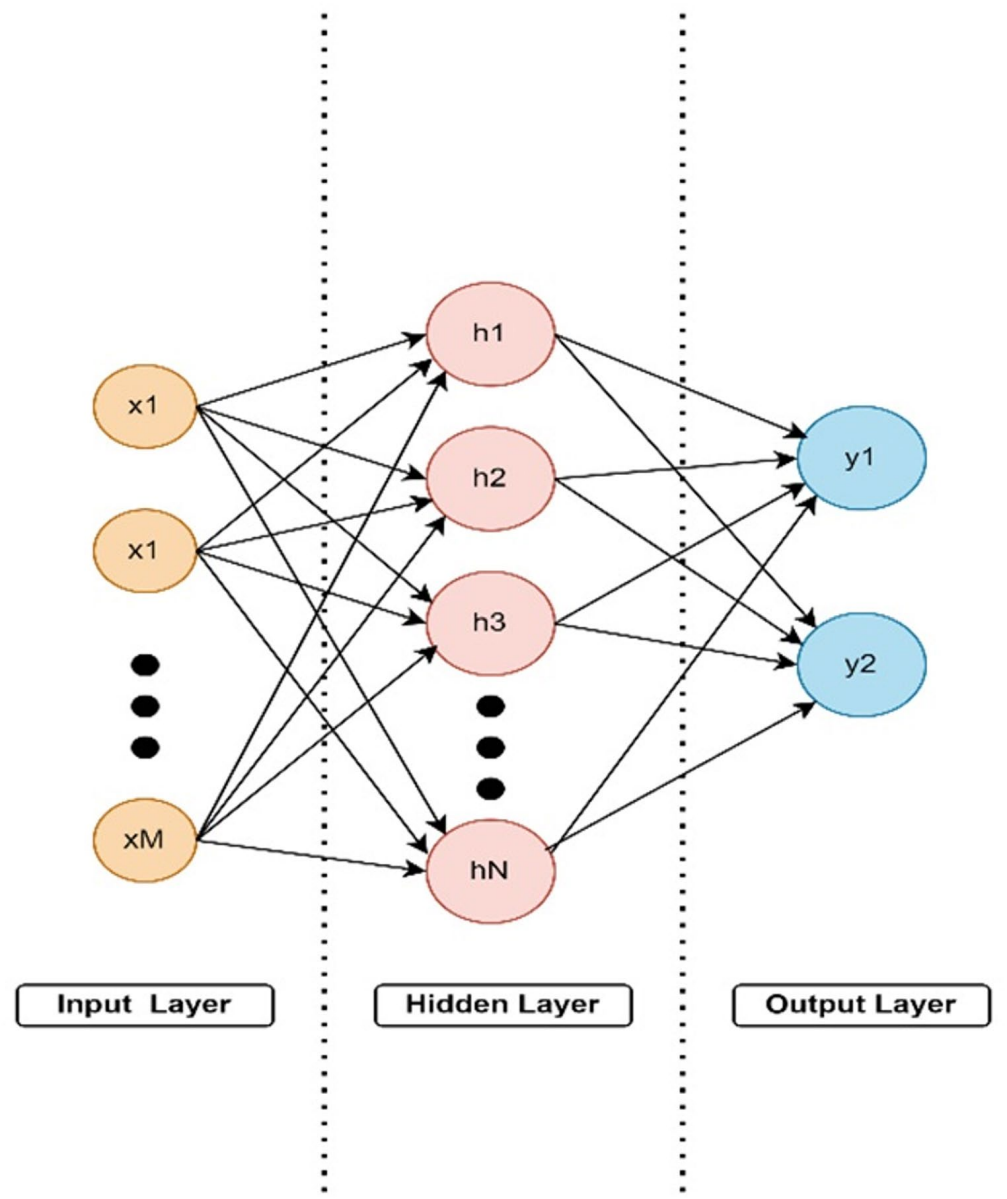
An example of ANN Structure

Artificial Neural Networks (ANN) is a machine learning model developed by mimicking the working principle of neurons in the human brain. This model is a powerful tool to solve complex problems in datasets efficiently and quickly. MLP-ANN [39] is a supervised neural network algorithm for classifying nonlinear models. The architecture of this algorithm consists of an interconnected input layer, hidden layer, and output layer, as shown in Fig. 6. Feedforward and feedback propagation are applied between these layers to perform the learning process in which weights and biases are automatically updated.

The formulas in Eq. (11) usually represent classification with the MLP-ANN algorithm. Where $\:\theta\:$ represents the weights, $\:x=\{x1,\:x2,\:x3,\:...\:xM\}$ the feature vector, $\:y$ the target vector, $\:b$ the bias term, and $\:\sigma\:$ the activation function.

11 $$\:y=f\left(x\right)=\sigma\:(\sum\:_{i=1}^{M}{x}_{i}.{\theta\:}_{i}+b).$$

### Training procedure and performance metrics for classifiers

**Fig. 7:**
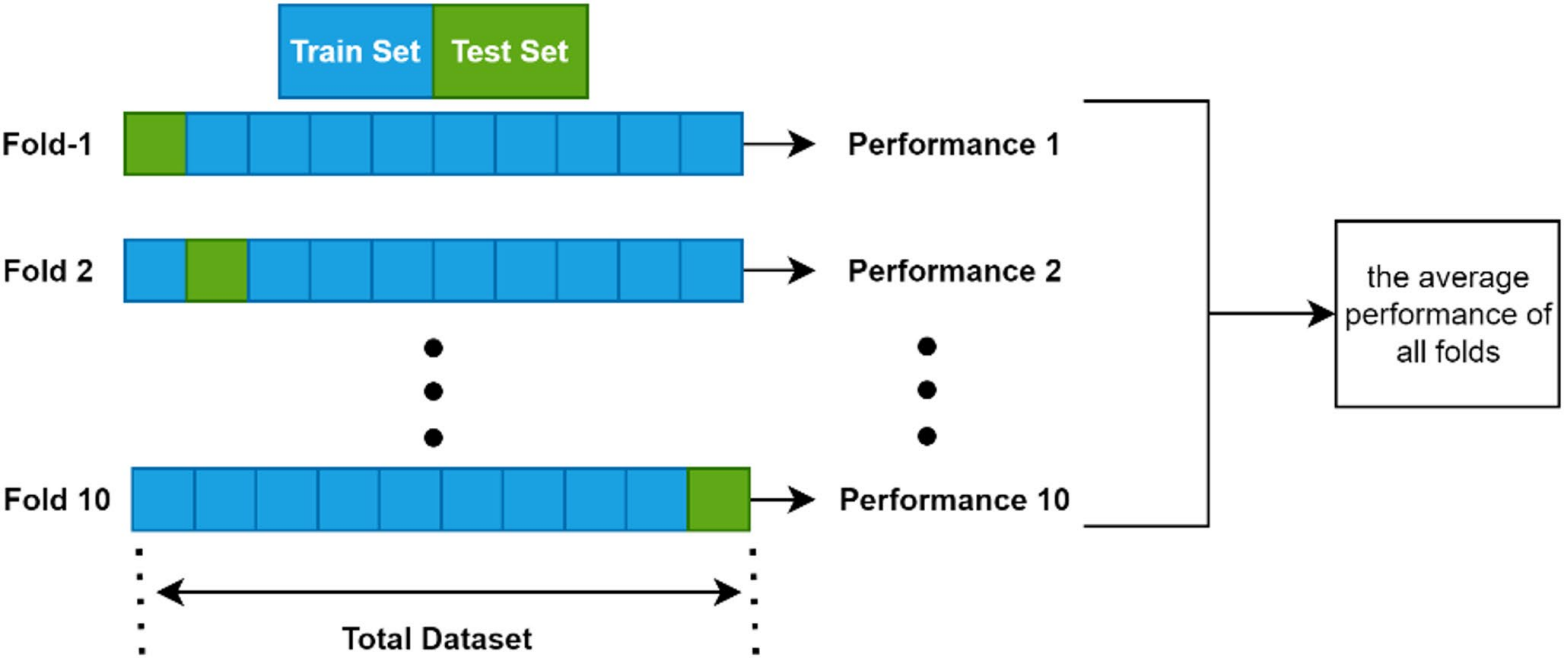
The diagram of k-fold cross-validation with k = 10

The performance of SVM, NB, LR, and ANN classifiers was evaluated using the k-cross validation method due to the limited dataset and to avoid overfitting. This method divides the dataset into k subsets during training and uses each subset as the training and test set. The model’s performance is evaluated for a different subset at each iteration. At the end of this process, k-test results are generated, and the average value of the results gives information about the model’s performance. In this study, the 10-fold cross-validation method was repeated 20 times to evaluate the performance of the classifiers more reliably (Fig. 7). Accordingly, for each fold, the dataset was split into training and test sets in a 9:1 ratio. Repeating the entire cross-validation process 20 times helped to minimize performance variability due to random data partitioning and ensured more stable and statistically reliable evaluation metrics. The performance metrics, including sensitivity, precision, accuracy, F1 score, specificity, and receiver operating characteristic area under the curve (ROC-AUC) score, obtained in each iteration, were averaged to minimize the effect of random variation in the training and test sets.

In this proposed work, to classify the dataset more efficiently and solve complex problems in this set more straightforwardly, the linear kernel function is used in the SVM algorithm, and the regularization parameter C is set to 1. For NB, class distributions were calculated using equal prior probabilities according to the number of samples of classes in the dataset. Different iteration numbers (100, 500, 1000, and 1500) were tried for the LR algorithm, and 1000 iterations were used to compare the best classification result with other algorithms. For MLP, two hidden layers with 10 nodes each were used, and the learning rate was set to 0.001.

The metrics used in this study to evaluate the performance of classification algorithms are precision, recall, accuracy, F1_score, specificity, and ROC-AUC score. Precision is a metric that expresses the ratio of correct and positive predictions to total positive predictions among the instances in the dataset. Recall is calculated as the ratio of correct and positive predicted instances to all true instances. Accuracy is the ratio of correctly predicted samples to the total amount of data in the dataset. The F1_score is a performance criterion for machine learning, which is the harmonic means of precision and accuracy. Specificity is a performance metric that measures the model’s ability to correctly identify negative samples by measuring the ratio of true negatives among all true negative samples. The ROC-AUC score is a metric that measures the classifier’s ability to distinguish between different classes by evaluating the relationship between the true positive rate and the false positive rate. The mathematical equations for precision, recall, accuracy, F1_score, and specificity are shown below:

12 $$\:Precision=\frac{TP}{TP+FP\:}$$

13 $$\:Recall=\frac{TP}{TP+FN}$$

14 $$\:Accuracy=\frac{TP+FN}{TP+TN+FP+FN}$$

15 $$F1\_Score = 2 \times ({{Sensitivity \times Precision} \over {Sensitivity + Precision}})$$

16 $$\:Specificity=\frac{TN}{TN+FP}.$$

Here, TP represents true positive samples, FP represents false positive samples, TN represents true negative samples, and FN represents false negative samples.

## Experimental results

In this section, we present a comparison of the amplitude and latency parameters obtained from MfERG responses of healthy and retinitis pigmentosa subjects. We also report results for binary and multiclass classification of retinitis pigmentosa using machine learning models including LR, NB, SVM, and MLP-ANN. Our approach is the first study to evaluate MfERG amplitude and latency parameters and use them for automatic staging of this disease.

However, to implement the experimental framework, different software tools were used at various steps of the work. In this regard, the feature extraction and visualization steps in the study were performed using MATLAB. In addition, the results of classification processes and performance metrics were obtained using the Scikit-learn library in the Python programming language.

### Comparison of MfERG responses of subjects

**Fig. 8:**
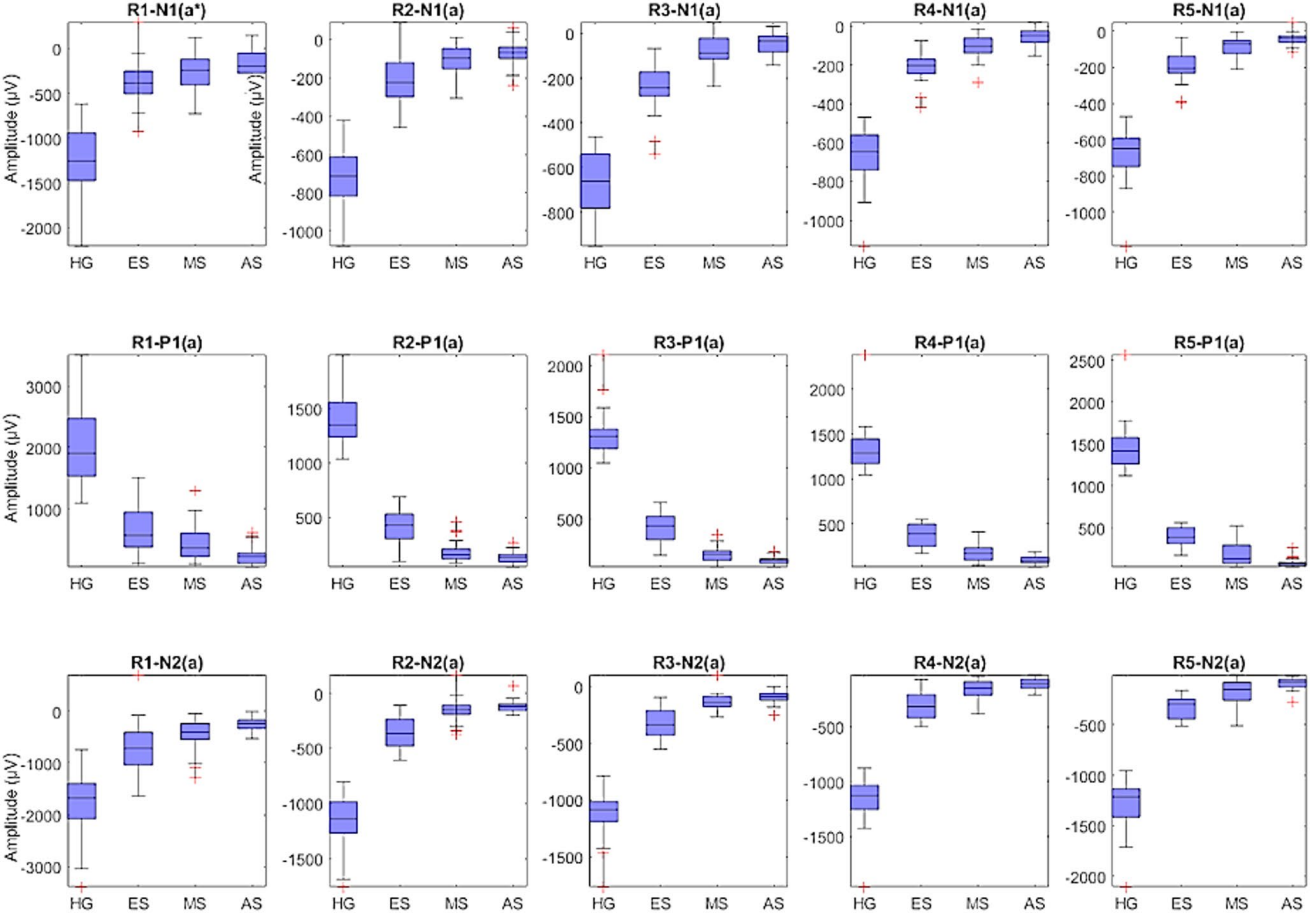
Comparison of 15 amplitude features for healthy group (HG), early stage (ES), mid-stage (MS), and advanced stage (AS)

**Fig. 9:**
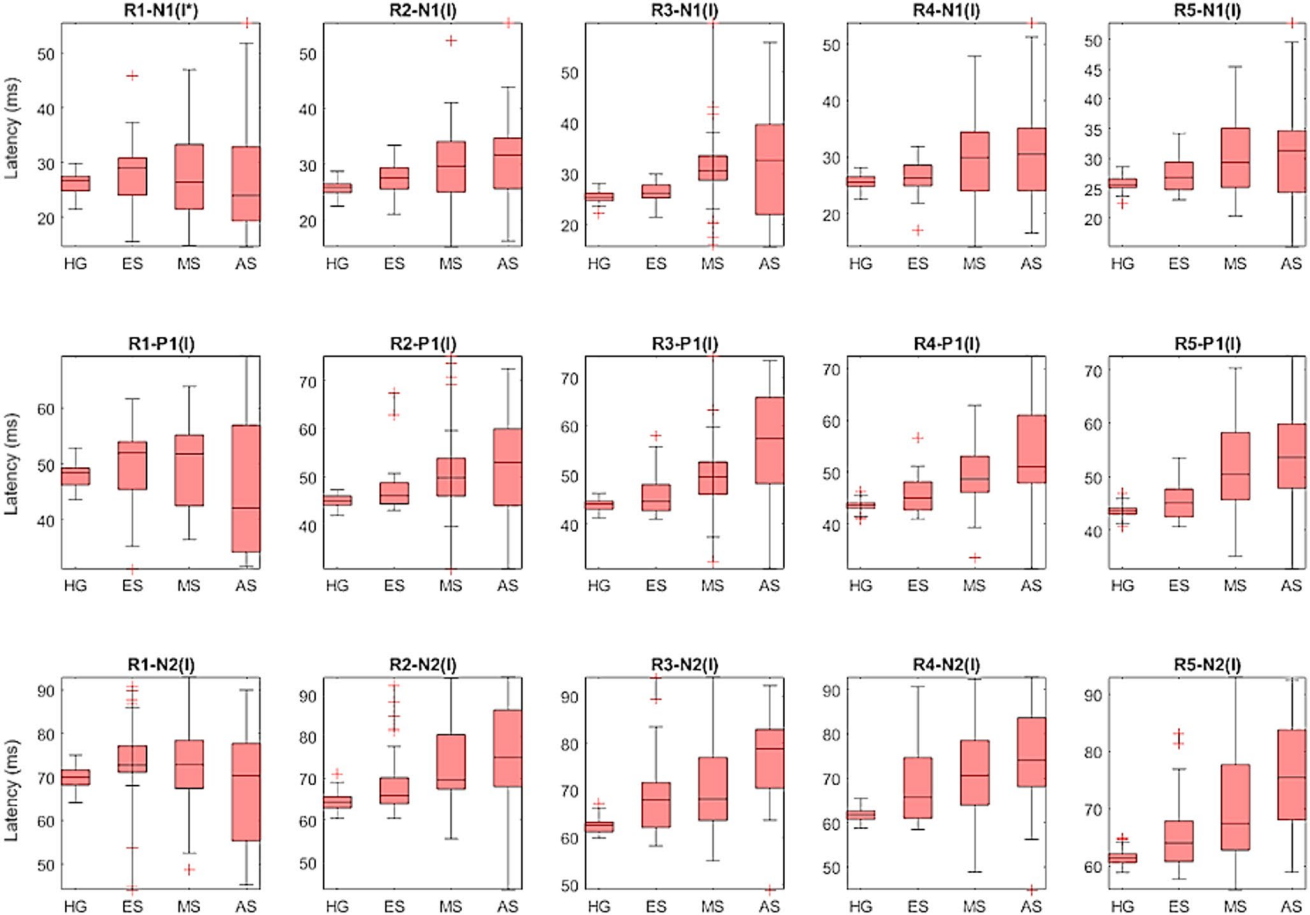
Comparison of 15 latency features for healthy group (HG), early stage (ES), mid-stage (MS), and advanced stage (AS)

In this proposed study, five rings (R1, R2, R3, R4, R5) are created using the MFERG responses and the commonly used amplitude and latency features N1, P1 and N2 are extracted for each ring. Since each ring has three amplitude and three delay features, a total of 15 amplitude and 15 latency features were extracted. These amplitude and latency features were visualized and compared for the healthy, early, mid and advanced groups using the box plots in Figs. 8 and 9. Accordingly, healthy individuals had higher N1, P1 and N2 wave amplitudes and shorter N1, P1 and N2 wave latencies than retinitis pigmentosa patients. Moreover, advanced stage showed relatively lower amplitude and higher latency in all rings compared to early and mid-stage. These results suggest that the amplitude and latency characteristics of N1, P1 and N2 waves are the most discriminative features for classifying healthy individuals and retinitis pigmentosa stages.

### Binary classification results

**Fig. 10:**
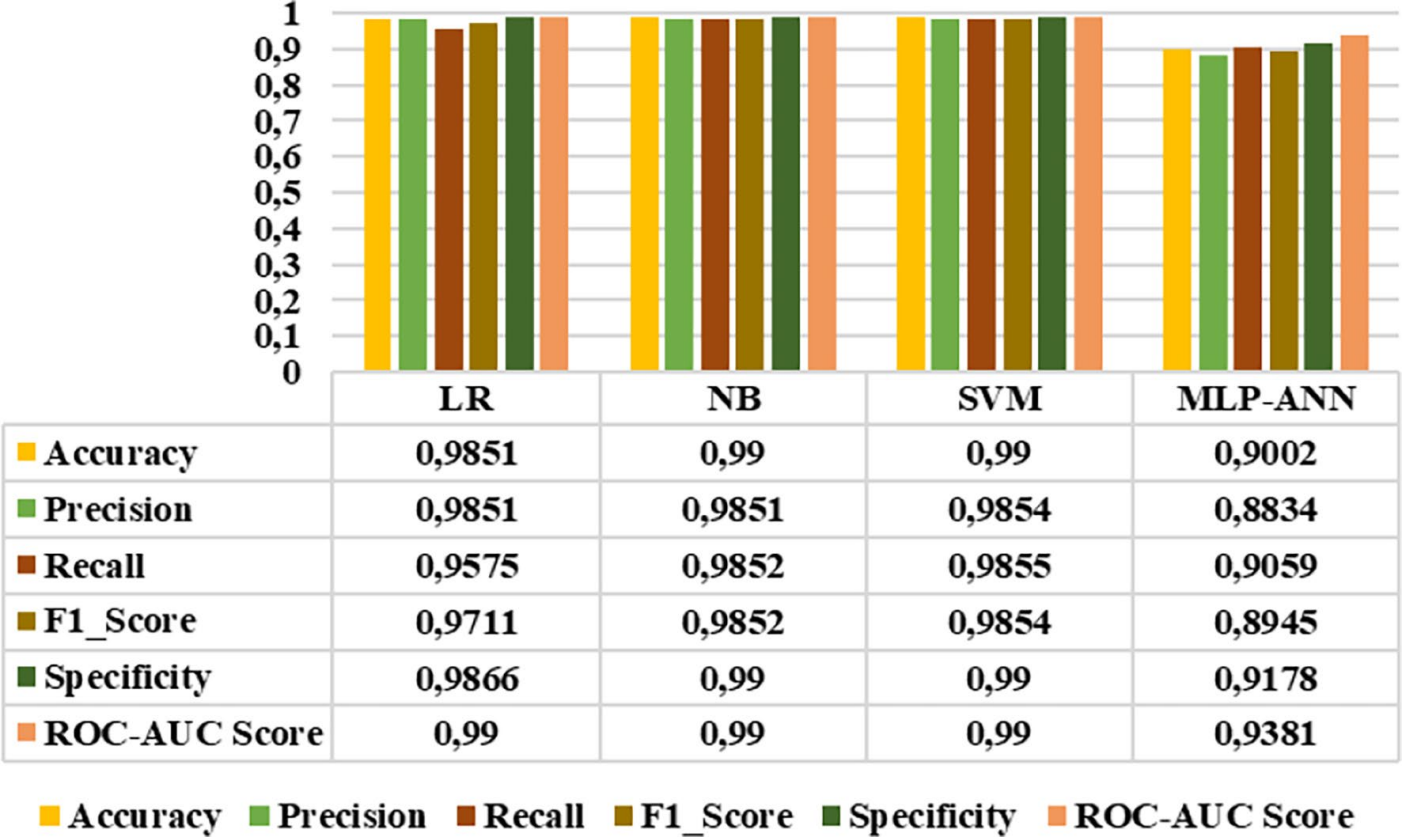
Healthy group vs. early-stage classification results

**Fig. 11:**
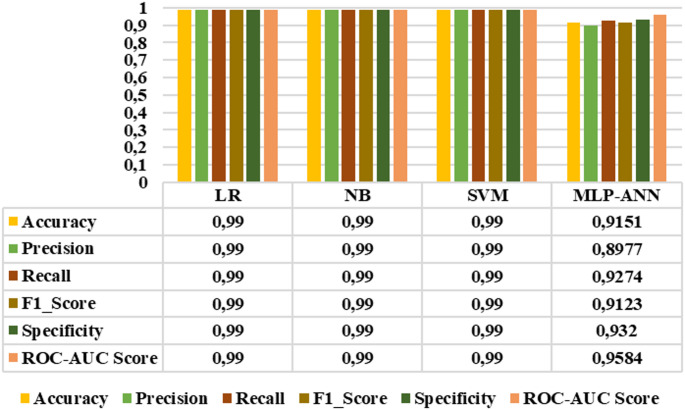
Healthy group vs. mid-stage classification results

**Fig. 12:**
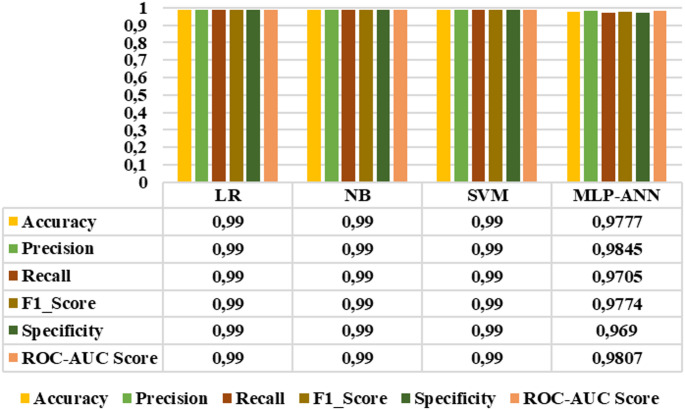
Healthy group vs. advanced stage classification results

**Fig. 13:**
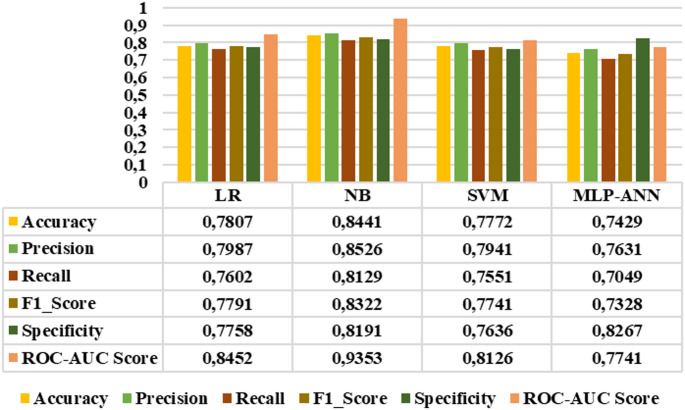
Early vs. mid-stage classification results

**Fig. 14:**
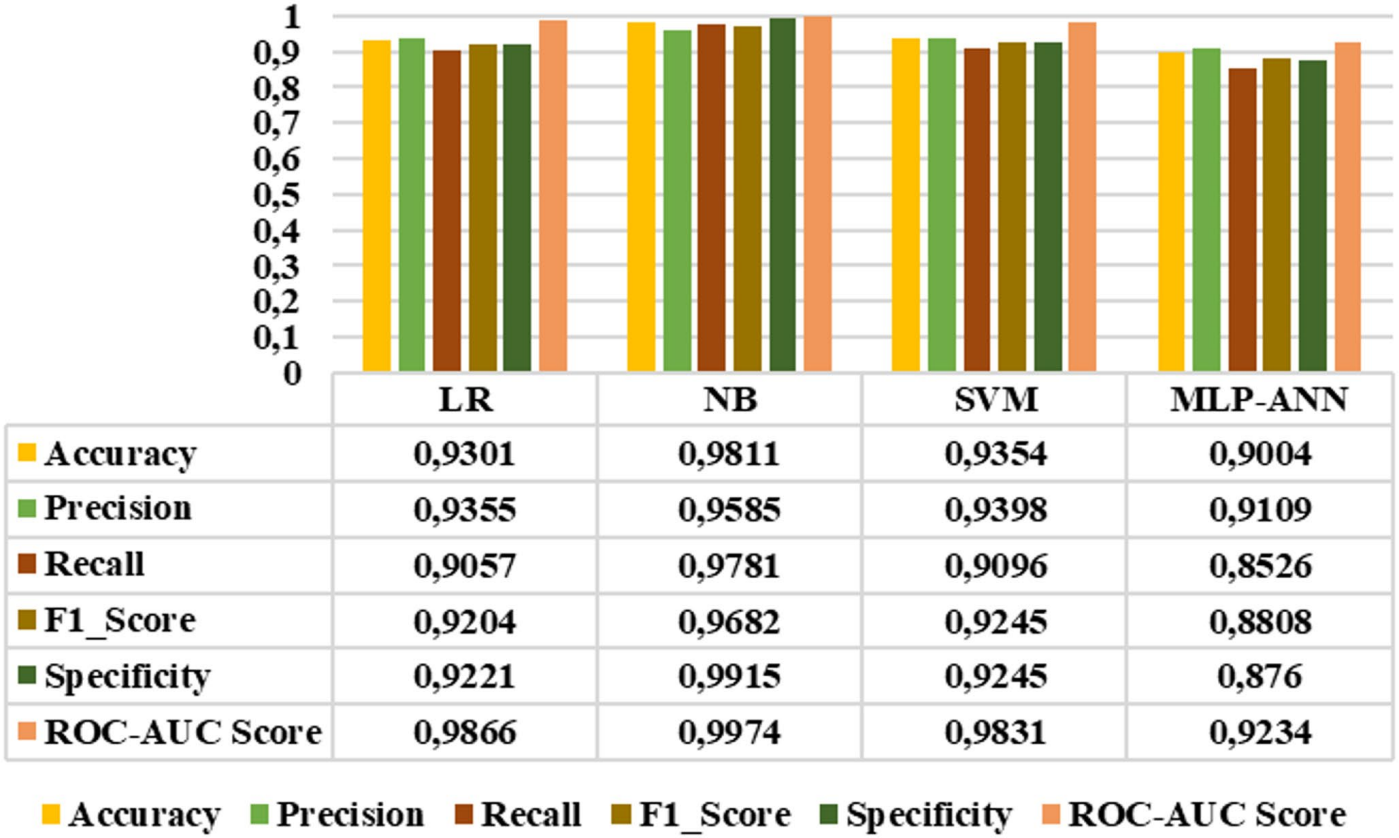
Early vs. advanced stage classification results

**Fig. 15:**
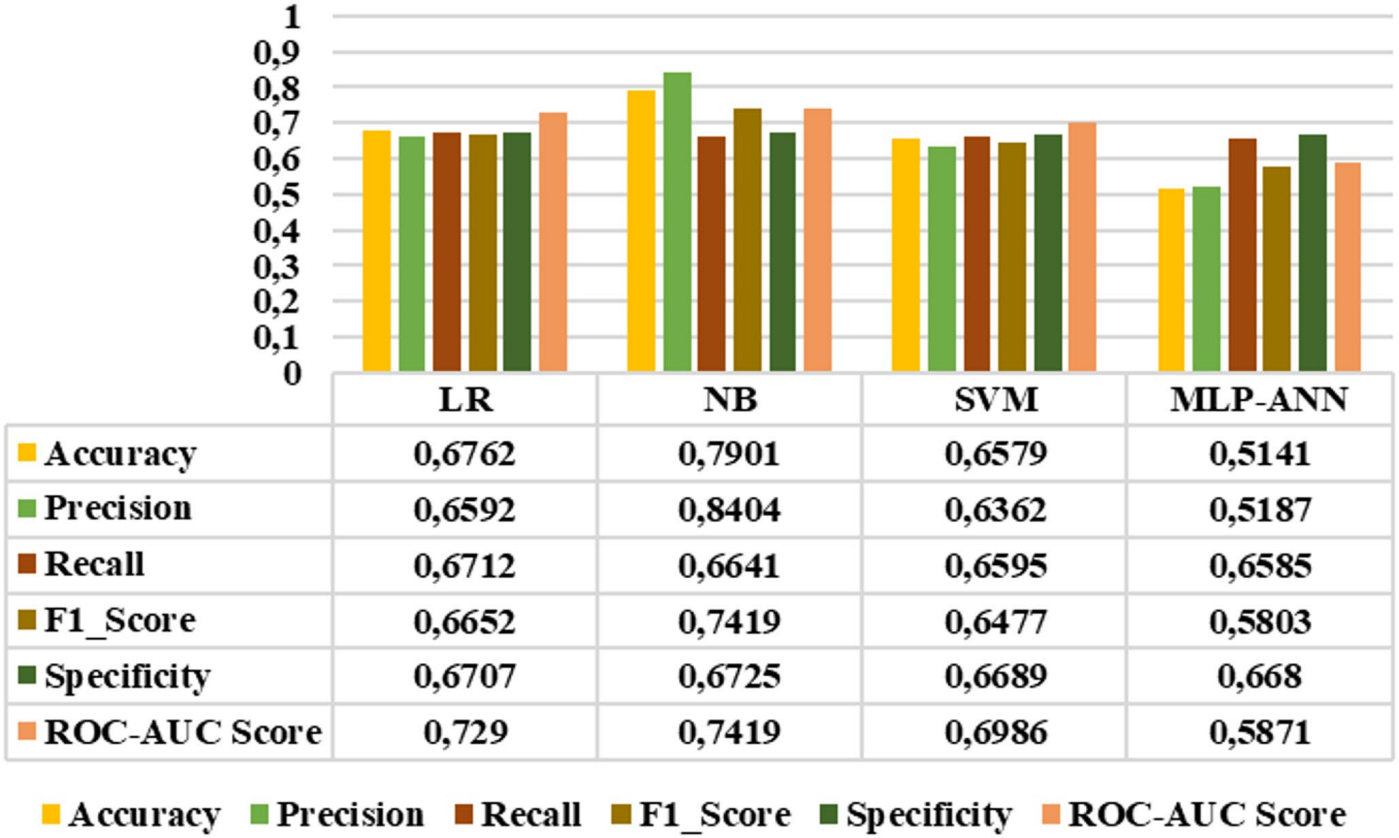
Mid vs. advanced stage classification results

Since all extracted features demonstrated distinctive characteristics, they were subsequently selected as inputs for LR, NB, SVM, and MLP-ANN classifiers. The performance of these classifiers was evaluated using accuracy, precision, sensitivity, F1_score, specificity, and roc-auc score metrics. Figures 10-15 demonstrated the performance metrics results for six classification types, namely: healthy group vs. early stage, healthy group vs. mid-stage, healthy group vs. advanced stage, early stage vs. mid-stage, early stage vs. advanced stage, and mid-stage vs. advanced stage. Compared to other classifiers, the NB algorithm achieved the highest accuracy rates of 0.99, 0.99, 0.99, 0.8441, 0.9811, and 0.7901 for the six classification types, respectively. This algorithm also gave the highest results in terms of precision, sensitivity, F1-score, specificity, and roc-auc score among all classification types. The results revealed that the proposed features and classification algorithms, especially NB and SVM, were effective in accurately discriminating healthy individuals from retinitis pigmentosa stages in binary classification tasks. Furthermore, within the retinitis pigmentosa group, classification between early and mid-stages and between early and advanced stages achieved higher results compared to other mid- and advanced-stage classifications.

### Multi-class classification results

In this study, in addition to binary classification tasks, multi-class classification tasks were performed to evaluate the effectiveness of the proposed features in discriminating between healthy individuals and retinitis pigmentosa stages. Two classification steps were designed: a four-class classification including the healthy group, early stage, mid-stage and advanced stage, and a three-class classification including only retinitis pigmentosa stages (early, mid and advanced).

**Fig. 16:**
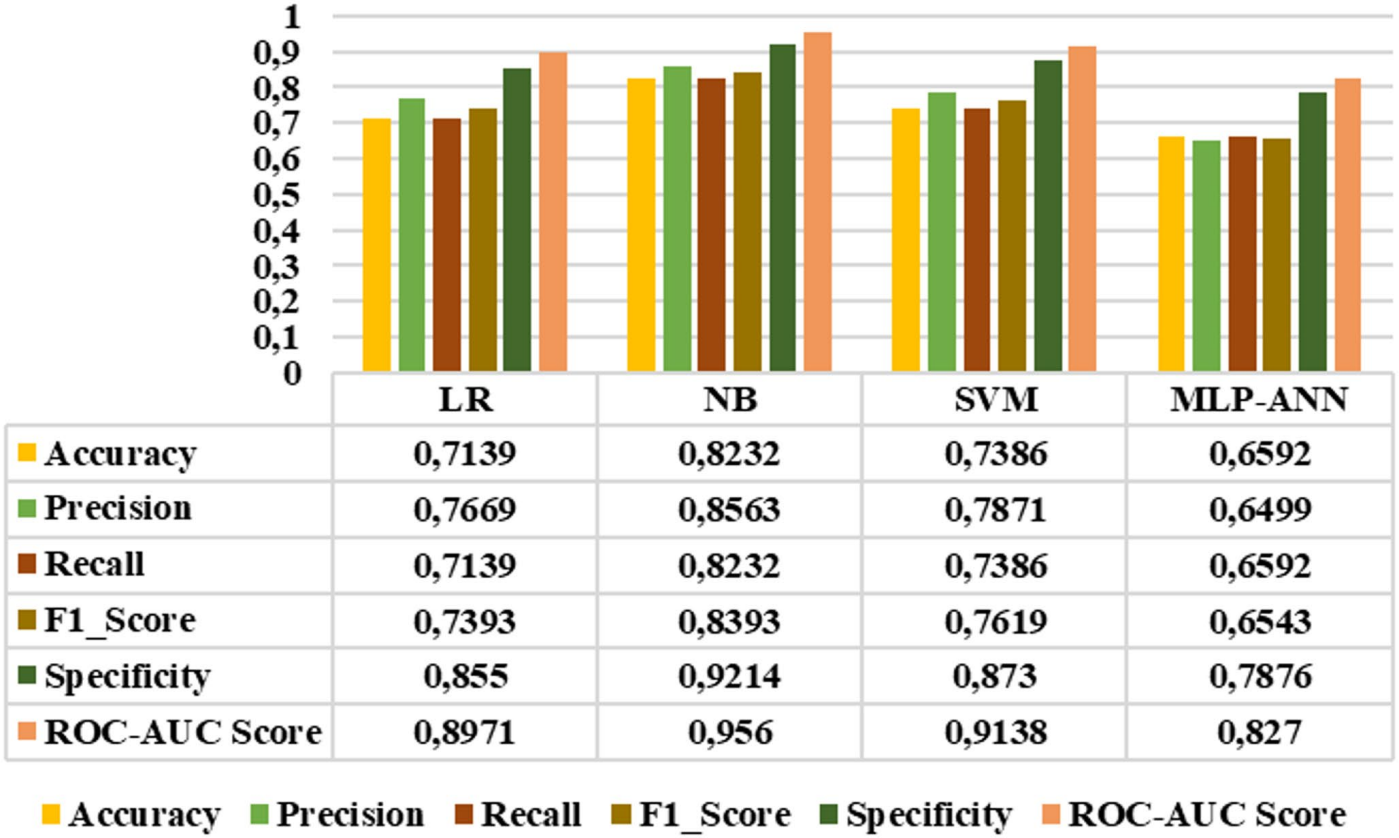
Four-class classification results

**Fig. 17:**
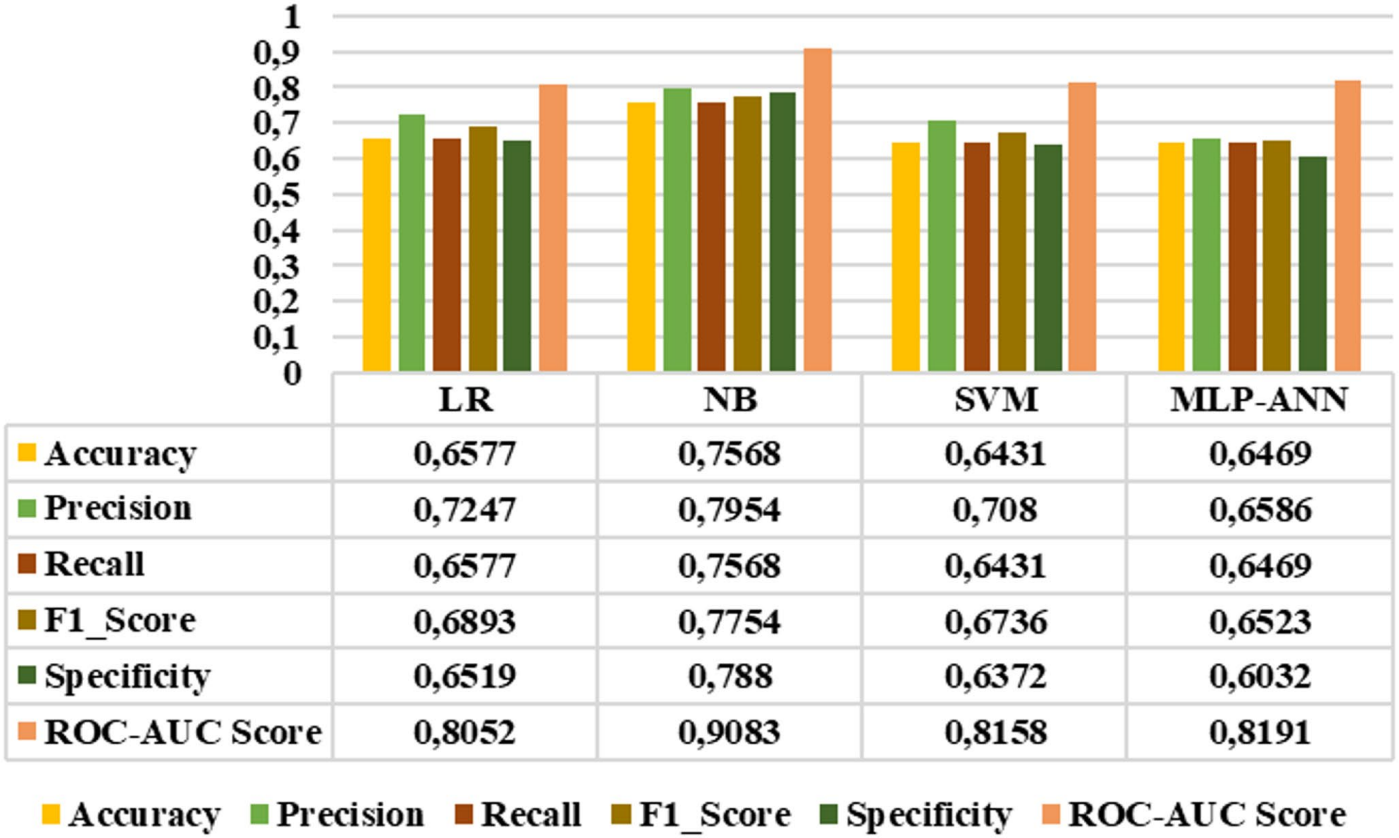
Three-class classification results

Figure 16 presents the results of the LR, NB, SVM, and MLP-ANN models for the four-class staging task, including the healthy group, early stage, mid-stage, and advanced stage categories. According to the graph, the NB model outperformed the other classifiers in all metrics, achieving the highest overall performance with an accuracy of 0.8232, precision of 0.8563, recall of 0.8232, F1-score of 0.8393, specificity of 0.9214, and roc-auc score of 0.956. On the other hand, Fig. 17 displays the classification results for the three-class staging task focusing only on retinitis pigmentosa stages (early, mid, and advanced stages), excluding the healthy group. This exclusion is based on the high separability of healthy individuals observed in binary classification tasks. In this classification step, NB achieved the highest scores in all evaluation metrics with an accuracy of 0.7568, precision of 0.7954, recall of 0.7568, F1 score of 0.7754, specificity of 0.788, and roc-auc score of 0.9083.

## Discussion

Electrophysiological tests, used to provide an objective assessment of the visual system, are fundamental elements of clinical practice. These tests provide important information for the diagnosis and monitoring of diseases by measuring electrical activities in different parts of the eye. Previous research has shown that the time and frequency domain features of MfERG responses, one of these electrophysiological tests, can be used to assess various diseases. For example, in the study by Boquete et al., a method based on neural networks was proposed to determine glaucoma by extracting 13 morphological features from the time domain of MfERG responses. It has been stated that the use of these features has provided effective results in successfully distinguishing individuals with glaucoma from the control group [40]. Brandao et al., on the other hand, analyzed the responses of two flash MfERG using discrete wavelet transformation to distinguish glaucoma from healthy controls, and the results were reported to be statistically significant [41]. Boquete et al., investigated the ability of MfERG responses to diagnose early-stage multiple sclerosis (MS). For this, they analyzed the amplitudes and latencies of MfERG and generated a signal from control individuals. As a result, they stated that the parameters of MfERG are significant in distinguishing between MS and healthy controls [35]. In another study, Lopez-Dorado et al. extracted features by applying Empirical Model Transform (EMT) and Continuous Wavelet Transform (CWT) to MfERG responses and used these features to classify controls and MS [42]. The above studies show that the attributes of MfERG responses have significant potential. In light of these, we have developed a machine learning model for early diagnosis and staging of retinitis pigmentosa disease using temporal features extracted from MfERG responses for the first time. Our results show that the model is successful in classifying this disease and can help in staging the disease.

The aim of this study is to present an efficient method to classify individuals with retinitis pigmentosa into both binary and multiclass stages using time features (amplitudes and latencies) of MfERG responses in combination with machine learning algorithms. However, several studies have examined various parameters of MfERG responses to assess outer retinal function in healthy subjects with retinitis pigmentosa. When these studies were reviewed, Han et al. found that only the mean amplitudes and latencies of N1, P1, and N2 waves of the R1 and R2 rings were decreased in retinitis pigmentosa compared to healthy individuals [14]. On the other hand, Giambene et al. used the amplitude and latency parameters of the mean P1 wave from MfERG responses to compare generalized retinitis pigmentosa, sector retinitis pigmentosa, and healthy individuals. They reported that the amplitude of the mean P1 wave was reduced and the latency was delayed in generalized retinitis pigmentosa compared to sector retinitis pigmentosa and healthy individuals [12]. As seen in these studies, retinitis pigmentosa and healthy individuals can be differentiated by different parameters of MfERG responses. In this article, we analyzed MfERG responses in the time domain to evaluate healthy subjects and retinitis pigmentosa stages. In accordance with ISCEV standards [13], we extracted all features of MfERG responses (amplitudes and latencies of N1, P1 and N2 waves of R1, R2, R3, R4 and R5). We observed a significant decrease in the amplitudes and a significant increase in the latencies of N1, P1, and N2 waves of all rings in the stages of retinitis pigmentosa compared to healthy groups (Figs. 8 and 9). These results are consistent with previous studies and confirm that retinitis pigmentosa reduces the field of view.

However, a previous study reported that MfERG is useful in advanced stages of retinitis pigmentosa disease [43]. In addition, Moon et al. divided patients with this disease into three different groups according to OCT images and measured the mean amplitude and latency parameters of N1 and P1 in rings 1 and 2. In this study, they found that these parameters were significantly different between the three groups [44]. In this study, we analyzed the amplitudes and latencies of N1, P1, and N2 waves in all rings between the stages of retinitis pigmentosa. Among these stages, Advanced stages showed relatively lower amplitude and higher latency in all rings compared to early and mid-stages. We can consider our results as close to the findings in the literature. In conclusion, in this study, all amplitude and latency features of the N1, P1, and N2 waves of the rings (30 features in total) were included in the investigation due to their high discriminative ability in distinguishing both individuals with healthy groups and retinitis pigmentosa and the stages of this disease.

When the studies on retinitis pigmentosa staging in the literature so far were examined, it was observed that various structural and functional techniques were used for staging in the studies of Iftikhar et al. [25] and Oner et al. [26]. Especially, as emphasized in the studies of Öner and Kahraman, MfERG parameters gave successful results in its staging. However, this approach is time consuming because it is manual and involves multiple techniques. In this study, we present a feasible approach for early diagnosis and its staging by analyzing the rings generated from MfERG responses and using them with machine learning algorithms.

In recent years, studies based on machine learning algorithms focusing on different imaging methods in patients with retinitis pigmentosa have gained attention in the literature. For example, in the study by [22], it has been reported that an automatic alignment technique based on AI, utilizing multimodal imaging techniques in individuals with retinitis pigmentosa, was successful compared to the manual alignment technique. In [23]’s study, the thickness of retinal layers in OCT images was automatically segmented using a deep learning architecture. In addition, in [24, 45], and [46], deep learning based detection of pigment markers in fundus images for the analysis of retinitis pigmentosa was performed and high performance was achieved for the its diagnosis. In two other studies, visual function in individuals with retinitis pigmentosa was successfully predicted using deep learning architectures [47, 48]. These studies show that imaging techniques and machine learning algorithms have significant potential for the analysis of retinitis pigmentosa. However, remarkably, these studies did not focus on tests that objectively assess the retina and provide functional measurements. Thus, in the current study, we used MfERG responses to determine the stage of retinitis pigmentosa, which provides a more detailed examination of retinal function.

As a result of the literature review, three studies were identified that applied machine learning algorithms to automatically distinguish between healthy individuals and those with retinitis pigmentosa. The machine learning results of these studies are compared with our results in Table 3.

**Table 3:** Comparison of the proposed method with similar studies in literature

References	Methods	Targets	Classifier	Results
Chen [19]	Color Fundus Image	RP/HG	Xception	0.96 ACC
				0.9571 SEN
				0.9851 PREC
Masumoto [20]	UWPC, UWAF	RP/HG	CNN	
				UWPC: 0.99 SPE,
				0.99 SEN
				UWAF: 0.99 SPE,
				1.0 SEN
Iadanza [21]	Pupillometry	RP/HG	SVM	0.846 ACC
				0.937 SEN
				0.786 SPE
Proposed Method	MfERG Responses	Binary Classification	NB	
		HG/ES, HG/MS, HG/AS, ES/MS		HG/ES: 0,99 ACC
		ES/AS, MS/AS		HG/MS: 0,99 ACC
				HG/AS: 0,99 ACC
				ES/MS: 0,84 ACC
				ES/AS: 0,98 ACC
				MS/AS: 0,79 ACC
			NB	
		Multi-class Classification		HG/ES/MS/AS:
		HG/ES/MS/AS		0.82 ACC
		ES/MS/AS		ES/MS/AS:
				0.75 ACC

RP: Retinitis Pigmentosa; HG: Healthy Group; ES: Early Stage; MS: Mid Stage; Advanced Stage; ACC: Accuracy; SEN: Sensitivity; PREC: Precision; CNN: Convolution Neural Networks; SVM: Support Vector Machines; NB: Naïve Bayes

As seen in Table 3, Chen et al. [19] classified retinitis pigmentosa and healthy individuals using color fundus images with high accuracy using Xception deep learning network. Masumoto et al. [20] obtained high success rates by classifying UWPC and UWAF images of these two groups with CNN. Similar to these two studies, our study successfully differentiated healthy individuals from retinitis pigmentosa. We used a total of four machine learning classifiers (LR, NB, SVM, SVM, MLP-ANN) and achieved the highest performance with the NB algorithm. In addition, Iadanza et al. [21] classified retinitis pigmentosa and healthy subjects using pupillometry parameters with SVM algorithm and found relatively lower success rates compared to other studies.

As can be seen, studies in literature have focused on distinguishing between retinitis pigmentosa and healthy individuals using machine learning algorithms, but so far, no specific method for its automatic staging has been presented. In this study, we not only distinguished individuals with retinitis pigmentosa from the healthy group but also performed both binary and multi-class classification to stage the disease. According to Table 3, NB, the algorithm with the best results classified the early vs. middle stage, early vs. advanced stage and middle vs. advanced stage with an accuracy of 0.84, 0.98 and 0.79 respectively. On the other hand, as observed in the same table, the NB classifier achieved an accuracy of 0.82 in the four-class classification that included healthy individuals, whereas it yielded an accuracy of 0.75 in the three-class classification, which involved only the stages of retinitis pigmentosa. The results show that different stages of retinitis pigmentosa disease can be effectively classified using temporal features of MfERG responses.

In summary, the proposed model demonstrates that individuals with healthy individuals and retinitis pigmentosa and stages of it can be effectively diagnosed from the N1, P1, and N2 amplitude and latency attributes calculated from 5 rings derived from MfERG responses. In addition, the practical extraction of these features may make this model applicable to a broader range of patients in clinical settings.

### Conclusion and clinical significance

Retinitis pigmentosa is a hereditary disease caused by damage to the cone and rod cells in the retina. This disease is relatively common in regions like Türkiye where consanguineous marriages occur. Its early diagnosis and correct staging are of great importance to slow or prevent the progression of the disease. Currently, there is no definitive cure for retinitis pigmentosa disease, but various approaches such as pharmacologic, gene- and stem-cell therapies are used to reduce the effects of the disease and delay its progression. In the [6] study, it has been emphasized that these treatment approaches may vary across different stages of retinitis pigmentosa. For example, pharmacologic therapies can be applied in the early stage, stem cell therapies in the middle stage and gene therapies in the advanced stage. These therapies have the potential to positively affect the course of the disease. In this context, Oner and Kahraman [49] investigated the effect of suprachoroidal umbilical cord-derived mesenchymal stem cell implantation on individuals with pediatric retinitis pigmentosa. As a result of the evaluations, significant improvement was observed in VA, VF and MfERG recordings (P1 amplitudes of ring 1, 2, 3, 4, 5). Özmert and Arslan [50] reported that VF, OCT, VA and MfERG (P1 amplitude and latency for ring 1, 2 and 3) measurements showed significant improvement by applying wharton gel-derived mesenchymal stem cell therapy for retinitis pigmentosa patients. On the other hand, a study by Mangunsong et al. [51] examined the effect of secretome injection derived from allogeneic umbilical cord mesenchymal stem cells on patients with AS-retinitis pigmentosa. In this study, the authors observed only a slight change in the N1 and P1 amplitudes and latencies of ring 1. In another study, Özkan et al. [52] investigated the effect of suprachoroidal implantation of mesenchymal stem cells in patients with retinitis pigmentosa and found significant improvement in VF, VA and MfERG (P1 amplitude and latency for ring 1, 3, 4) tests. These studies show that treatment modalities are effective, which plays an important role in improving the quality of life of retinitis pigmentosa patients. Therefore, it is crucial to accurately diagnose the stages of the disease and determine treatment strategies.

The main advantage of this study is that not only healthy and retinitis pigmentosa individuals but also the stages of this disease can be binary and multi-class classified using the basic parameters of MfERG responses. Therefore, the stages of retinitis pigmentosa disease have been proven to be associated with MfERG. On the other hand, the limitation of the present study is that this disease is hereditary, and the data are limited to individuals with retinitis pigmentosa from Türkiye. In conclusion, for the first time, we have taken an essential step towards early diagnosis and staging of retinitis pigmentosa disease by creating a feature vector with temporal parameters of MfERG responses and successfully using machine learning algorithms.
